# SMART-COP score for patients admitted with community-acquired pneumonia (CAP) to an ICU in a district general hospital: a smarter way of identifying patients with severe CAP?

**DOI:** 10.1186/cc12075

**Published:** 2013-03-19

**Authors:** M Pachucki, F Kelly, A Padkin

**Affiliations:** 1 Frenchay Hospital, Bristol, UK; 2 RUH, Bath, UK

## Introduction

British Thoracic Society guidelines on community-acquired pneumonia (CAP) advocate ICU referral for patients with CURB65 score of 4 and 5. A recently developed scoring system, SMART-COP, designed to identify patients at need of intensive respiratory or vasopressor support (IRVS), has been validated in a variety of settings. It predicts the need for ICU admission (defined as need for IRVS) with greater accuracy than CURB65, but is not used routinely in our UK institution.

## Methods

We retrospectively analysed critical care admissions of patients with a diagnosis of CAP in a UK district general hospital - ICNARC-coded diagnoses of pneumonia (bacterial, viral, no organisms isolated) over a 7-month period (August 2011 to January 2012). We ascertained the CURB65 and SMART-COP scores on referral to the ICU and matched them in relation to the need for IRVS, length of inotropic and ventilatory support and ICU length of stay.

## Results

Our search revealed 28 potential matches. Five patients were excluded (not CAP) and the notes for seven patients were not available for analysis. We analysed the notes of 16 patients matching our criteria. In this small sample, there was a strong association between increasing SMART-COP score and the need for IRVS (correlation coefficient *r *= 0.96). There was also a strong correlation with longer inotropic support (*r *= 0.85) and longer ventilatory support (*r *= 0.96) with increasing SMART-COP scores but a weaker correlation with length of ICU stay (*r *= 0.49). Moreover, none of the patients admitted to the ICU had CURB65 score higher than 3 at the time of ICU referral.

## Conclusion

In our small sample, higher SMART-COP score was associated with increased likelihood of IRVS. This suggests that a further study with a larger sample size should be performed to investigate whether SMART-COP is an improvement on CURB65 in predicting the need for IRVS in UK intensive care patients.
